# How young pedestrians do explain their risky road crossing behaviors? A qualitative study in Iran

**DOI:** 10.15171/hpp.2017.26

**Published:** 2017-06-14

**Authors:** Mina Hashemiparast, Reza Negarandeh, Ali Montazeri

**Affiliations:** ^1^Department of Public Health, Maragheh University of Medical Sciences, Maragheh, Iran; ^2^Nursing and Midwifery Care Research Center, School of Nursing and Midwifery, Tehran University of Medical Sciences, Tehran, Iran; ^3^Health Metrics Research Center, Institute for Health Sciences Research, ACECR, Tehran, Iran

**Keywords:** Pedestrian, Risky road crossing behaviors, Young adults, Qualitative study

## Abstract

**Background:** Although pedestrian-vehicle accidents are often the results of risky behaviors during road crossings, there is limited evidence concerning the risky road crossing behaviors of pedestrians. This study was aimed at eliciting and exploring the reasons that can help explain why young pedestrians take risky road crossing behaviors.

**Methods:** A qualitative content analysis approach was conducted on purposefully selected young individuals who had the experience of vehicle-collision accident. Data collected by in depth, semi-structured interviews until data saturation and concurrently analyzed, assisted by MAXQDA 10.

**Results:** Three main categories emerged as social reasons for risky road crossing behaviors of the young pedestrians including ‘conformity with the masses/crowds’, ‘lack of social cohesion and sense of belonging in social relations’ and ‘bypassing the law/ law evasion’.

**Conclusion: ** The risky road crossing behaviors of young pedestrians are found influenced by the pedestrian attitudes towards the political, social, cultural and economic condition of the society.Moreover, popular culture and collective behaviors in crossing the roads influenced the risky behaviors. Hence, personal, cultural and social interventions could be effective in promoting the young pedestrians’ behavior.

## Introduction

Pedestrians are one of the most victims of car accidents especially in countries with poor road traffic laws and policies to improve pedestrian safety.^1^ Annually many pedestrians face injury and death from road traffic accidents and road fatalities account to 22% of the pedestrians’ globally.^2^

Based on the World Health Organization (WHO) report on pedestrian fatalities in Iran about half of those killed in accidents are pedestrians and motorcyclists.^3^ The pedestrian road traffic injuries and fatalities are associated with indirect costs such as psychological disorders, depression and high economic burden.^4,5^ The pedestrian-vehicle collision event is a multidimensional social and public health phenomenon.^6^ Several factors related to the environment, vehicle and human are involved in the event process.^7^ Although pedestrians are vulnerable and more at risk than other road users (such as car drivers or car occupants), the illegal and unsafe road crossing behaviors of the pedestrians usually lead to the crashing accidents.^8^ The accidents could mainly be related to the pedestrian’s behavioral factors that could largely be prevented by interventions that could modify the personal and social behaviors.^9^

According to the road traffic police in Tehran, 38% of pedestrian crashes were related to risky road crossing behaviors.^10^ The potential victims are the young adults who are usually present on all roads and want to cross the streets regardless of the traffic signs.^11^ Although the road traffic accident rate among young adults (18-25 years old) in Iran is very high,^12^ many studies focused on pedestrians’ behavior of older people and children.

The risky road crossing behaviors of pedestrians are subject to context specific factors. In most of the quantitative studies, the reasons for the risky road crossing behaviors of pedestrian are not reported. Hence, the influence of cultural and contextual factor on the risky behavior of pedestrians is still not clearly known. A thorough understanding about the risky road crossing behaviors of pedestrians in each cultural context can contribute to effective accident prevention and reduction among at risk population groups. The study was aimed at eliciting and exploring the reasons that can help explain why young pedestrians take risky road crossing behaviors.

## Materials and Methods

### Study design & participants

A qualitative study design using conventional content analysis approach was conducted to elicit and explore why young pedestrians take risky road crossing behaviors.

Purposeful sampling method with maximum variation in terms of gender and geographic areas of Tehran was used. The participants were selected from young people who had experienced vehicle-collision accident and qualified to participate in the study because of having first-hand experience about the event. The age of 18 to 25 years old, resident of Tehran and willingness to participate in the study, were the inclusion criteria. The details about the participants are presented in Table 1.

### Data collection 

Data collected by in-depth, semi-structured interviews. The interviews performed individually and usually began with a general question, e.g. ’could you please explain your experience of car- accident?’ ‘What was your role in that vehicle-collision?’ ‘How would you describe road-crossing experience in streets of Tehran?’ Further explanations were also obtained based on the responses of the participants and by asking complementary probing questions such as ‘Would you please elaborate on your explanation?’ ‘Explain more please!’

The interviews were recorded using a digital voice recorder and by taking field notes of the interviews. The time and place of the interview sessions were arranged based on the convenience of each the interviewee. Hence, the places for interview were in the park, participant’s workplace, and subway station in Tehran. The interview for each participant lasted between 60 and 90 minutes. The interview was continued until the data saturation, a point where no new concepts or ideas were emerged. Accordingly, data saturation was reached by interviewing a total of 12 individuals (10 boys and 2 girls).

### Data management and analysis

The data analysis was performed concurrently with the data gathering. All the interviews were transcribed verbatim immediately and to gain the general impression, each interview was read several times before the analysis. Then, the data were broken down into units of meaning that were extracted from the statements and labeled with conceptual names (codes). The interviews coded sometimes using *in vivo* coding technique. The codes were compared based on constant comparative method (similarities and differences) and grouped into categories as suggested by Graneheim and Lundman.^13^ MAXQDA 10 software was used to manage the textual data during the coding process.

### Data trustworthiness 

This study applied the criteria suggested by Guba and Lincoln in several ways to evaluate the credibility of the data.^14^ Sampling with maximum‏ variation approach helped to increase the credibility of the data. This sampling strategy provided different perspectives about the phenomenon under the study. The prolonged engagement with the participants during the interview period helped to establish trust and better understanding of the participants. Moreover, the research team checked the interview data and findings at each step of the study process. The researchers documented all the steps followed in the research process to provide auditability and dependability of the data.

## Results

This study provided a new perspective about the reasons for the risky road crossing behaviors of the young pedestrians in Tehran. Three main reasons for young pedestrians’ risky road crossing behaviors were described in three main categories: ‘conformity with the masses/crowds’, ‘lack of social cohesion and sense of belonging in social relations’ and ‘bypassing the law/law evasion’. Each of these categories is extensively discussed as follows:

### Conformity with the masses/crowds

The young pedestrians believed that conformity with the masses/crowds was one of the main reasons for their risky road crossing behaviors. That is, they believed that they have adopted the road crossing behavior from other pedestrians. This can be understood from the expression of one of the participants who states:

“…When I found that other pedestrians crossed the street in potential risky situations, I also did the same and crossed the street like them.”

Another participant holds that anomie and chaos are becoming a norm and assumed that people form their behaviors based on such a condition. In explaining the idea the participant said:

“…to be honest, I am supposed to wait until the car pass if I have to cross the street. If I wanted to behave like others, the priority of crossing is mine. But when many pedestrians are not waiting until the cars finished passing, why I should do.”

Disobeying the traffic regulations on crossing roads and observing other pedestrians’ road crossing behaviors seems a potential emotional factor that encouraged the pedestrians to behave the same way when crossing roads in the study area.

According to the participants, perception, not internalizing the traffic rules and regulations are the main reasons for the anomie and chaos in road crossing behaviors. This is reflected in the ideas of one of participants who expressed:

“Traffic norms have not been internalized by pedestrians; this is why traffic norms are ignored. If traffic related institutions could help with internalizing traffic norms, pedestrians never ignored the ‘Don’t cross sign’ and would use traffic equipment such as pedestrians’ bridges.”

Furthermore, most of the participants believed that neighborhood could influence pedestrians’ road crossing behaviors. The pedestrians’ behaviors in one areas of the city were different from those in the other side of the same city. The pedestrians in downtown areas tended to cross the roads in potential risky situations. The disobedience of traffic regulations in such areas was considered a normal behavior. In support of this idea one of the participants said:

“At the upper side of the city, if a pedestrian wanted to cross the street, s/he waits until all the cars pass. However in the downtown, people ignore the ‘Don’t cross sign’ because this illegal and risky road crossing is considered a normal behavior.” 

### Lack of social cohesion and sense of belonging in social relations

The participants mentioned that lack of patience due to reduction in social trust was one of the reasons for the risky road crossing behaviors of pedestrians. The lack of social trust‏ and sense of belonging to social relations was believed result in ignoring the rights of others. Such a sense of distrust was perceived as leading to ignoring the traffic laws and regulations. The participants expressed this situation as:

“…the distrust that we have in our relationship with one another is translated into our behavior as pedestrians. Everyone including the pedestrians and drivers thinks that it is his/her priority to cross the road.”

Another participant also added that the main reason for the potential risky road crossing behaviors was a lack of social trust in the peoples’ relationships. This was expressed in the participant’s word as:

“…behind the unsafe road crossing behaviors, there is a huge distrust among people in the society. No one trusts the others and this is natural, because people are trying to deceive others. A lack of social trust is the main issue.”

Other participants also believed that ignoring the rights of pedestrians and failure to adhere to the norms of the society contributed to the risky road crossing behaviors. This phenomenon rooted in the degradation of social rights at the macro level. This perspective is described as follows:

“… I believe that the high-ranking individuals spoil their rights. Then, they also spoil others’ rights. We just share this belonging to the society. I think the spoiling our rights are the main issue.” 

### Bypassing the law/law evasion

The tendency to disobey the traffic rules and regulations that rooted in the cynicism and distrust of the police was another reason for risky road crossing behaviors.

Most of the participants mentioned that the incompetency and ineligibility of the police as another reason for the failure to obey the laws and practicing the unsafe behaviors against the traffic laws. One of the pedestrians said:

“…. when I see a policeman who is oblivious/ reckless to the anti-social behaviors such as substance abuse, I lose my trust to the policeman. I always disagree with such a police. And I do not like to be forced more than I can tolerate.”

Another participant also confirmed the same point and said: *when the police didn’t obey to the law, why I should do.*

## Discussion

The study explored the reasons for the risky road crossing behaviors of the young adults who had the experience of vehicle-collision accident. One of the most important reasons identified for the risky road crossing behaviors was conformity with masses/crowds.‏ The participants believed that disobeying the traffic regulations by other pedestrians have influenced their road crossing behaviors. The respondents matched their road crossing behaviors with a group norms and rules.

Hence, obeying or disobeying of the road traffic laws by other pedestrians played an important role in making decisions whether to cross the road or not in potentially risky situations‏. Earlier studies also reported that pedestrians were more likely to behave in a manner other individuals behave. A study in Iran found that density of the population affected the decisions of pedestrians on using the pedestrian bridges. That is the pedestrian tended to cross the street without using the pedestrian bridge when others did so.^15^ Similarly, a study in China reported that the pedestrians’ tendency to follow others’ road crossing behaviors against the traffic signals.^16^ The social psychologists also confirmed that the pedestrians’ road crossing practice could influence others’ road cross behavior as there is the possibility of someone to follow what everyone else does. Moreover, the young adults participated in our study felt the presence of some sort of disorder and chaos in the society. They believed that non-compliance with the regulations was a common behavior in the Iranian civic culture and they matched their road crossing behaviors with such a social disorganization and anomie. Similarly, earlier study in Iran reported the presence of a relationship between the feeling of anomia and non-compliance to traffic regulations.^17^ The disobedience to the traffic regulations in our study were perceived rooted in the cultural context of neighborhood. The social behaviorists believe that human beings learn behaviors from the environment they have the exposure.^18^

Hence, the culture of the urban areas can affect the pedestrians’ road crossing behaviors. Evidence also showed the presence of a relationship between illegal road crossing behaviors and living in impoverished areas.^19^ Our study also indicated that the‏ distrust and loss of familiarity in social relationships could lead people to ignore the rights of each other. They behaved in a manner that set their personal and individual interests in life priority rather than the collective interests.

This finding is consistent with the concept of social capital that holds a system of social norms that can enhance the level of cooperation among people.^20^ Social capital can be seen as a phenomenon that is affected by social institutions, human relationships and norms.^21^ In societies with high social capital, the crime and violations of laws and norms of the society is less and it can be measured by the level of social trust.^22^

Trust is an important factor in sustaining and improving social relationships. The lack of trust among members of a society can hinder social orders and interactions.^23^

Therefore, increasing the interests and sense of belongingness to others can increase the adherence to social norms and establishes order and social cohesion.^24^ The tendency to illegality and legal transgressions were also among the reasons for risky road crossing behaviors among young people. This lawlessness is a situation in which many individuals deliberately overlook the community norms. The practices may be consisted of a range of behaviors such as permanent escape from the law including violent and non-violent theft to occasional law escape such as running against the red lights and ignoring traffic rules and regulations.^25^ The participants’ distrust and skepticism about the perpetrators of the traffic police in our study were the reasons for their disrespect of the law. This might be consistent with the concept of “protesting illegality”. This illegality was perceived when the legislative bodies were not considered legitimate. Thus, the people did not obey the laws and deliberately break them. Legality or illegality by people in a society is affected by the attitude of the citizens towards the government and legislative bodies in the society. Negative attitudes toward the law enforcement, lack of trust, unjust discrimination and inappropriate implementation of the laws and rules are the underlying causes of illegality.^26^ This can be affected by multiple social, political, cultural and economic factors. The inefficiency of the police in dealing with others’ moral dissonance in the society such as drug dealing in recreational areas or parks and public places were other reasons for the illegality in our study. When the enforcement of the laws does not have the necessary competence to implement the law, the citizens will develop mistrust towards the laws and ultimately attempt to escape from them. Civil distrust is the most important factor that affects the attitude of people in a society. If the law enforcements are perceived as not respecting people, the people who are expected to respect the laws will be reluctant to comply.^25^

### Limitations

The in-depth interview for two of the participants was carried out through telephone call because it was not convenient for them to avail in person for the interview.

## Conclusion

This study attempted to explain the social reasons for risky road crossing behaviors of young pedestrians in Tehran. The risky road crossing behaviors of the young pedestrians were influenced by their attitudes towards the political, social, cultural and economic condition of Iran. Moreover, popular culture and collective behaviors in crossing the roads influenced the risky behaviors of the young pedestrians. Several strategies at personal, cultural and social level could be effective in promoting young pedestrians’ road crossing behaviors in Tehran.

## Ethical Approval

The ethics committee of Tehran University of Medical Sciences (TUMS) approved the study protocol. Written informed consent was obtained from each participant after explaining the aim and process of the study. The participants were informed that they had the right to withdraw at any time during the interview process or the study. The interviews were recorded performed anonymously using code number assigned to the interviewees.

## Competing interests

The authors have no conflict of interest to declare.

## Authors’ contributions

This was a part of PhD thesis of the first investigator (MH). AM supervised the development of work. RN was the study advisor. All authors read and approved the final version.

## Funding

Tehran University of Medical Sciences (TUMS) provided financial resources.

## Acknowledgements

The authors wish to appreciate all participants for their involvement in the study.

**Table 1:** Demographic profile of participants (n = 12)

**Variable**	
Age (y)	21.9 ± 3.39
Marital status	
Single	11(91.67)
Married	1(8.33)
Education	
Primary	1(8.33)
Secondary	4(33.33)
Higher	7(58.34)
Job	
Student	3(25.0)
Employee	8(66.67)
Soldier	1(8.33)

Data were presented using mean ± SD for age and No. (%) for categorical variables.

## References

[1] Schwebel DC, McClure LA, Severson J (2014). Usability and feasibility of an internet-based virtual pedestrian environment to teach children to cross streets safely. Virtual Real.

[2] Hamed MM (2001). Analysis of pedestrians’ behavior at pedestrian crossings. Saf Sci.

[3] World Health Organization. Global status report on road safety 2013: supporting a decade of action. Geneva: World Health Organization; 2013.

[4] Gitelman V, Balasha D, Carmel R, Hendel L, Pesahov F (2012). Characterization of pedestrian accidents and an examination of infrastructure measures to improve pedestrian safety in Israel. Accid Anal Prev.

[5] Ghorbani B, Hakim AS, Zare k. Epidemiologic study of fatal traffic accidents in Khuzestan province in 2010. Journal of Rescue & Relief 2012; 4(2):28-35. [Persian].

[6] Rosenbloom T (2009). Crossing at a red light: Behaviour of individuals and groups. Transp Res Part F Traffic Psychol Behav.

[7] Lund IO, Rundmo T (2009). Cross-cultural comparisons of traffic safety, risk perception, attitudes and behaviour. Saf Sci.

[8] Yagil D (2000). Beliefs, motives and situational factors related to pedestrians self-reported behavior at signal-controlled crossings. Transp Res.

[9] Gordon H (2004). Psychiatry, the law and death on the roads. Adv Psychiatr Treat.

[10] Razzaghi A, Salehi A, Heidari K, Zolala F (2014). Exploring the barriers and facilitators in using of pedestrian bridges among pedestrians: a qualitative study. Safety Promotion and Injury Prevention.

[11] Diaz EM (2002). Theory of planned behavior and pedestrians’ intentions to violate traffic regulations. Transp Res Part F Traffic Psychol Behav.

[12] Saffarzadeh M, Hasanpour S, Abdi A (2011). Analysis of pedestrian accidents in Iran. Road Journal.

[13] Graneheim UH, Lundman B (2004). Qualitative content analysis in nursing research: concepts, procedures and measures to achieve trustworthiness. Nurse Educ Today.

[14] Guba E, Lincoln Y. Fourth Generation Evaluation. Newbury Park, CA: Sage Publications; 1989.

[15] Soltani A, Mozaiyani S. Investigation of factors affecting willingness to utilize pedestrian bridge. Journal Of Geography and Planning 2010;15(32):95-124. [Persian].

[16] Zhou R, Horrey WJ, Yu R (2009). The effect of conformity tendency on pedestrians’ road-crossing intentions in China: an application of the theory of planned behavior. Accid Anal Prev.

[17] Abbasszadeh M, Abedini I, Hasani MR, Bakhshayesh M (2014). Social anomia and disobeying traffic regulations. Security and Social Order Strategic Studies Journal.

[18] Ritzer G. Contemporary Sociological Theory and Its Classical Roots: The Basics. 3rd ed. New York: McGraw-Hill: 2009.

[19] Tulu GS, Washington S, King M, Haque M. Why are pedestrian crashes so different in developing countries? A review of relevant factors in relation to their impact in Ethiopia. Australasian Transport Research Forum 2013; Proceedings; October 2013; Brisbane, Australia.

[20] Moradian Sorkhkalaee M, Eftekhar Ardebili H, Nedjat S, Saiepour N. Social capital among medical Students of Tehran University of Medical Sciences in 2011. Razi Journal of Medical Sciences 2012;19(102):30-7. [Persian].

[21] Alvani M, Shirvani A. Social Capital (Concepts, Theories and Applications). Tehran: Mani publication; 2006. [Persian].

[22] Kennedy BP, Kawachi I, Prothrow-Stith D, Lochner K, Gupta V (1998). Social capital, income inequality and firearm violent crime. Soc Sci Med.

[23] Tajbakhsh K. Social Capital, Trust, Democracy and Development. 1st ed. Tehran: Shiraz publication; 2004. [Persian].

[24] Rafi Pour F. Anatomy of Society. 6th ed. Tehran: Enteshar Publications; 2011. [Persian].

[25] Babai A, Jaiyan F. The sociological analysis of effective factors on law evasion. Danesh-e-Entezami 2011;11(4):7-58. [Persian].

[26] Rezai M (2007). The frequency, type and factors affecting law evasion. Iranian Journal of Sociology.

